# Supplementary material for: Barriers to Early Presentation amongst Rural Residents Experiencing Symptoms of Colorectal Cancer: A Qualitative Interview Study
Source: Cancers (Basel). 2022 Dec 31;15(1):274. doi: 10.3390/cancers15010274 (PMC9818976; doi:10.3390/cancers15010274)
Supplement: Supplementary file 1 [file cancers-15-00274-s001.zip › cancers-2100704-Supplementary material.pdf]

Table S1: Participant Characteristics

Participant ID	Sex	Age Category	Rural Residence Category	"Native" or "Migrant" Resident	Index of Multiple Deprivation (1 = most deprived, 5 = least deprived)	Employment Status	Symptoms	Consulted about Symptoms?
P01	F	60-69	Rural Village	"Migrant" Rural Dweller	3	Retired	Diarrhoea, rectal bleeding, stomach pain	Yes
P02	F	60-69	Rural Village	"Migrant" Rural Dweller	3	Retired	Diarrhoea, Stomach Pain	No
P03	F	60-69	Rural Village	"Migrant" Rural Dweller	5	Retired	Diarrhoea, Stomach Pain	Yes
P04	F	50-59	Rural Village	"Native" Rural Dweller	5	Employed: Part Time	Diarrhoea, Constipation, Stomach Pain	No
P05	F	30-39	Hamlets & Isolated Dwellings	"Migrant" Rural Dweller	4	Unemployed	Diarrhoea, Constipation, Stomach Pain	No
P06	F	70-79	Rural Village	"Migrant" Rural Dweller	5	Retired	diarrhoea	Yes
P07	M	50-59	Hamlets & Isolated Dwellings	"Native" Rural Dweller	4	Self-Employed	Diarrhoea, Constipation, Stomach Pain	Yes
P08	F	60-69	Rural Village	"Migrant" Rural Dweller	5	Retired	Diarrhoea, Stomach Pain	Yes
P09	M	80-89	Rural Village	"Native" Rural Dweller	5	Retired	Diarrhoea, Constipation, Stomach Pain	Yes
P10	F	50-59	Hamlets & Isolated Dwellings	"Native" Rural Dweller	3	Self-Employed	Stomach Pain	No
P11	M	40-49	Rural Village	"Migrant" Rural Dweller	5	Employed: Full Time	Diarrhoea, Rectal Bleeding, Stomach Pain	Yes
P12	M	60-69	Rural Village	"Migrant" Rural Dweller	5	Retired	Constipation, Stomach Pain	No
P13	F	40-49	Rural Town and Fringe	"Native" Rural Dweller	5	Employed: Part Time	Constipation	No
P14	F	70-79	Rural Town and Fringe	"Native" Rural Dweller	5	Retired	Diarrhoea, Stomach Pain	No
P15	F	60-69	Rural Town and Fringe	"Native" Rural Dweller	3	Employed: Part Time	Diarrhoea, Constipation, Rectal Bleeding, Stomach Pain	No
P16	F	50-59	Rural Village	"Migrant" Rural Dweller	4	Unemployed	Diarrhoea, Stomach Pain	No
P17	F	50-59	Rural Village	"Native" Rural Dweller	4	Employed: Part Time	Constipation, Rectal Bleeding, Stomach Pain	No
P18	F	60-69	Rural Town and Fringe	"Native" Rural Dweller	5	Self-Employed	Constipation, Rectal Bleeding, Stomach Pain	Yes
P19	F	70-79	Rural Village	"Native" Rural Dweller	4	Retired	Stomach Pain	Yes
P20	F	80-89	Rural Town and Fringe	"Native" Rural Dweller	5	Retired	Constipation, Stomach Pain	Yes
P21	F	60-69	Rural Village	"Migrant" Rural Dweller	4	Employed: Part Time	Constipation	No
P22	F	50-59	Rural Village	"Native" Rural Dweller	4	Retired	Constipation	No
P23	M	60-69	Rural Town and Fringe	"Native" Rural Dweller	5	Retired	Constipation	Yes
P24	M	60-69	Rural Town and Fringe	"Migrant" Rural Dweller	2	Retired	Diarrhoea	No
P25	M	60-69	Rural Town and Fringe	"Native" Rural Dweller	2	Retired	Diarrhoea	No
P26	F	50-59	Rural Village	"Native" Rural Dweller	3	Employed: Part Time	Constipation, Stomach Pain	No
P27	F	70-79	Rural Village	"Migrant" Rural Dweller	3	Retired	Diarrhoea	No
P28	F	90-99	Rural Village	"Migrant" Rural Dweller	3	Retired	Constipation, Stomach Pain	No
P29	M	40-49	Rural Village	"Migrant" Rural Dweller	3	Employed: Full Time	Diarrhoea, Stomach Pain	No
P30	M	60-69	Rural Town and Fringe	"Native" Rural Dweller	5	Employed: Full Time	Constipation, Stomach Pain	No
P31	M	70-79	Rural Town and Fringe	"Native" Rural Dweller	5	Retired	Diarrhoea, Constipation, Rectal Bleeding, Stomach Pain	No
P32	F	60-69	Rural Village	"Migrant" Rural Dweller	3	Self-Employed	Diarrhoea	Yes
P33	F	70-79	Rural Village	"Native" Rural Dweller	3	Retired	Diarrhoea, Rectal Bleeding, Stomach Pain	No
P34	M	80-89	Rural Village	"Migrant" Rural Dweller	3	Retired	Constipation, Stomach Pain	Yes
P35	F	60-69	Rural Village	"Migrant" Rural Dweller	3	Retired	Constipation, Stomach Pain	Yes
P36	F	60-69	Rural Village	"Migrant" Rural Dweller	3	Self-Employed	Constipation, Stomach Pain	Yes
P37	M	70-79	Rural Village	"Migrant" Rural Dweller	3	Retired	Constipation, Rectal Bleeding	No
P38	M	40-49	Rural Village	"Native" Rural Dweller	3	Unemployed	Stomach Pain	Yes
P39	M	60-69	Rural Village	"Migrant" Rural Dweller	3	Self-Employed	Stomach Pain	No
P40	F	60-69	Rural Village	"Native" Rural Dweller	3	Self-Employed	Diarrhoea, Constipation, Stomach Pain	No

RURALLY

Recognition, Understandings of, and Responses to bowel symptoms Among people Living in rural Localities of Yorkshire

Interview Topic Guide

This is a topic guide, to inform the content of the interview and topics to be covered, however, there is flexibility in incorporating questions on novel topics, probing participants, and changing topic order, as best suits each individual interviewee.

Symptom Topics
In the survey you said you had experienced x symptom, could you tell me a bit more about it?

Points to cover:
- When it started
- When it ended (or still ongoing?)
- What they thought it was
- Had they experienced that symptom before
- How did they feel about the symptom (worried, not bothered etc)
- Did it impact their work activities and, if so, how?
- Did it impact their family responsibilities and activities and, if so, how?
- Did it impact other daily or leisure activities and, if so, how?
- Did they talk to anyone about it? GP, pharmacist, practice nurse, 111, A&E, family, friend etc
  - What was the other persons' thoughts about it?
- Did they go to GP or A&E about it?
  - If yes, date of consult

repeat for every symptom and ask if there were others they didn't mention in the survey

Accessing Primary Care Topics
- How long does it take them to travel to the GP surgery
- How do they typically get there? Car, bus, cycle, walk, taxi, other
- Do they find it easy to get an appointment?
- How do they feel about their local GPs and other staff at the practice?
- Are there other local health care services that they use? (eg pharmacy)
- Is there anything that would make it easier to go to the GP?

Rural Location & Daily Life Topics
- How long have they lived in this, and other, rural areas?
- What is their occupation?
  - If it's a rural occupation, how does that influence symptom appraisal and management. Seasonality?
- What are their living circumstances and caring responsibilities

- Do they have family living close by? Has the family lived in the area for generations?
- Do you think people who live in rural areas are healthier?
- What are some of the health benefits of living in a rural area?
- What are some of the drawbacks of living in a rural area?

- Would you say that there a different groups, or types of people who live in rural areas? How would you describe these different groups?
- Do you think these different groups think about health or illness differently?

- Do you think that rural people think about health and illness differently to how someone in a town or a city might?
- Do you think people in rural areas are better equipped to deal with illness than those living in a town or a city might be?
    - If so, how?
- Do you think that they use the GP services differently?
- Do you think that rural people have a different relationship with the GP, or GP surgery as a whole, than people in say, a city, might do?

- Do you think that there is anything that stops 'rural people' from going to the doctors at times?
- Can you think of anything that may make it easier for yourself, or other rural people, to go to the doctor's when they are unwell?

Post Pandemic Questions

Primary Care Consultations
Have you had any telephone/video/e-consult consultations with your GP, or a practice nurse, since the start of the pandemic, in relation to ANY symptoms?
YES OR NO
If so:

- Did you feel like you are able to adequately explain the problem?
- Did you find it easier, or more difficult, to discuss symptoms 'remotely', than you might have done in person?
    - Possible prompts – logistical ease of childcare, reducing exposure risk, being overheard by family/confidentiality, building rapport, distractions of home, script and performative nature of attending the doctor's
- Did you feel that you were able to talk about ALL of the symptoms that were concerning you, as well as you may have done at an in-person consultation?
- Do you feel that you are able to talk about symptoms that you see as possibly unrelated to your main symptom – does this differ from how comfortable you might feel to raise an additional problem during an in-person consultation?
- Do you think that these types of consultation work for all health conditions or are there certain types of symptoms that you feel like it would never be appropriate for?
        - Does this differ by different types of remote consultation?
- Do you feel like they (GP/practice nurse) are able to properly assess you over the phone/video?
    - Do you feel like the GP is able to do their job to the best of their abilities using remote consultations?
- Do you think that video/telephone consults could be used more in the future for people who live in rural areas?
- In terms of accessing GP services during the pandemic, what systems or new ways of accessing services, do you think worked well? What didn't work well?

Accessing Health Care Post-COVID

- Has Covid19 changed how you feel about going to the GP?
    - If so, how?
- Do you feel concerned about potentially exposing yourself (or your household) to COVID-19 by attending your GP surgery?
- Are there things that you would now no longer go to the doctor's about?
    - If so, do you think this is how you feel at the moment and it is likely to change in the future?
- Are there certain things you would always go to the doctor's about?
- Are there other places/people you would now consider going to for advice when you are unwell, before going to the doctor's?

- Has Covid19 changed how you would feel about potentially having to go to hospital?
    - If so, how?

- Have your thoughts and feelings about the NHS more broadly changed since the start of the pandemic, both in terms of NHS staff and the NHS as an organisation?
- Has the pandemic impacted your feelings about the trust you have for the NHS?
    - Does this trust differ between GPs and hospitals?

Post Pandemic Rural Life

- Do you think that COVID-19 has impacted rural communities in ways different to people living in say a city?
    - Impact on accessing services with public transport and remoteness
    - Impact on shopping with local shops closing
- Has it impacted the way the community interact?
    - Did you do any community activities before and how have these changed?
        - What does this mean to you?
- Do you think the pandemic will have a long term impact on rural communities?
        - In what ways?

Thank the participant and ask if there is anything else they would like to add about their thoughts on being a rural patient, and what that means when they are ill and for accessing health care?

RURALLY CODING FRAMEWORK

SYMPTOMATIC EXPERIENCE / THE PATIENT INTERVAL

Symptoms & Help-Seeking

Nature of symptoms
Impact of symptoms
Feelings towards symptoms
Managing symptoms (diet etc)
Length of symptoms
Considering a cause
Considering cancer
Discussing symptoms
Family history of symptoms
Help-Seeking Decision Making

- Sx's don't require a consultation
- Sx's remain 'too long'
- Initial explanations no longer hold

Consulting

Making an appointment
Consultation encounter
Secondary care investigations for symptoms
Barriers to consulting for sx's

- Distance
- Transport
- Time
- GP/Px relationship
- Finances
- Opening times

RURAL HEALTH CARE SERVICES

Primary Care

Mode of travel to GP
Distance from GP
Relationship with GP
Relationship with practice staff
Appointment triage
Appointment availability
Barriers to consulting for sx's
Distance
Transport
Time
GP/Px relationship
Finances
Opening times
Making consultation easier
'Wasting the Dr's time'

Specialist & Allied Health Svcs

Out of hours services
Pharmacies
A&E
Secondary care
Patient transport

INDEXING CODES

Resides in a village
Resides remotely
Always lived rurally
Rural migrant
Tendency towards infrequent consultation
Tendency towards 'regular' consultation

RURAL HEALTH & ILLNESS

Benefits of rurality

- Clean air
- Outdoor Living
- Less stress
- Peace & quiet
- Community
- Good primary care services

Disadvantages of rurality

- Distance to specialist services
- Public transport
- Withdrawal of services
- Isolation

Attitudes towards health

- Stoicism
- Self-sufficiency
- Active lifestyle
- 'getting on with it'

Managing illness in rural areas
Rural people as healthier
Rural people as less health
Health & rurality not related
Ageing in rural areas
Planning forwards to deal with future illness
Applying knowledge of illness & bodies learned from animal husbandry to own symptom & illness experiences
